# The Mediating Role of Team Resilience at Work Between Teamwork Practice Environment, Team Functioning and Cohesion in Oncology: A Cross-Sectional Study

**DOI:** 10.3390/curroncol33040232

**Published:** 2026-04-20

**Authors:** Dominique Tremblay, Djamal Berbiche, Susan Usher, Marie-José Durand, Kelley Kilpatrick, Marjolaine Landry, Sylvie Lessard, Thomas G. Poder, Catherine Prady, Mathieu Roy, Nassera Touati, Annie Turcotte

**Affiliations:** 1 Faculty of Medicine and Health Sciences, Université de Sherbrooke, Longueuil, QC J4K 0A8, Canada; djamal.berbiche@usherbrooke.ca (D.B.); marie-jose.durand@usherbrooke.ca (M.-J.D.); catherine.prady.med@ssss.gouv.qc.ca (C.P.); mathieu.roy7@usherbrooke.ca (M.R.); annie.turcotte@usherbrooke.ca (A.T.); 2 Centre de Recherche Charles-Le Moyne, Longueuil, QC J4K 0A8, Canada; susan.elizabeth.usher@usherbrooke.ca (S.U.); sylvie.lessard@usherbrooke.ca (S.L.); nassera.touati@enap.ca (N.T.); 3 Centre de Recherche de l’Hôpital Maisonneuve-Rosemont, Centre Intégré Universitaire de Santé et de Services Sociaux de l’Est-de-l’Île-de-Montréal, Montréal, QC H1T 2M4, Canada; kelley.kilpatrick@mcgill.ca; 4 Susan E. French Chair in Nursing Research and Innovative Practice, Montréal, QC H3A 2M7, Canada; 5 Faculty of Medicine and Health Sciences, Ingram School of Nursing, McGill University, Montréal, QC H3A 2M7, Canada; 6 Department of Nursing, Université du Québec à Trois-Rivières, Drummondville, QC J2C 0R5, Canada; marjolaine.landry@uqtr.ca; 7 Centre de Recherche du Centre Hospitalier Universitaire de Sherbrooke, Sherbrooke, QC J1H 5N4, Canada; 8 Faculty of Medicine, Université Laval, Québec, QC G1V 0A6, Canada; thomas.poder@fmed.ulaval.ca; 9 Centre de Recherche de l’Institut Universitaire en Santé Mentale de Montréal, Centre Intégré Universitaire de Santé et de Services Sociaux de l’Est-de-l’Île-de-Montréal, Montréal, QC H1N 3V2, Canada; 10 Centre de Recherche du CHU de Québec-Université Laval, Québec, QC G1V 4G2, Canada; 11 Centre Intégré de Santé et de Services Sociaux de la Montérégie-Centre, Greenfield Park, QC J4V 2G9, Canada; 12 École Nationale d’Administration Publique, Montréal, QC H2T 3E5, Canada

**Keywords:** oncology, team resilience, teamwork practice environment, team functioning, team cohesion, quantitative research

## Abstract

Oncology teams operate in highly demanding clinical environments marked by recurrent acute and chronic stressors that can impair optimal quality of care. Although the practice environment is known to influence team processes, the specific contribution of team resilience at work to team effectiveness remains insufficiently delineated in oncology. This cross-sectional study investigated whether team resilience at work mediates the associations between the teamwork practice environment and two core indicators of team effectiveness: team functioning and team cohesion. A total of 189 oncology team members in Québec (Canada) completed an e-questionnaire between February 2022 and June 2023. Structural equation modeling was conducted to assess an evidence-informed mediation model. The tested model revealed acceptable fit. Findings showed significant indirect effects consistent with a mediating role of team resilience at work in the relationships between the teamwork practice environment and team functioning and team cohesion. Some dimensions—resourcefulness, alignment, efforts to understand problems, wellness awareness and being proud to work in the team—loaded strongly on the resilience concept. These results highlight the relevance of reinforcing team resilience capacities to sustain high-quality care in oncology settings. Interventions aiming to enhance team effectiveness may benefit from explicitly integrating strategies designed to strengthen resilience-related dimensions within oncology teams.

## Simple Summary

Oncology teams work in fast-paced and high-pressure environments, where repeated challenges can make it challenging to maintain high-quality care. We examined whether team resilience at work—the ability to adapt, stay focused, and recover together—plays a role in the relationship between the teamwork practice environment and team functioning and cohesion. Oncology professionals in Québec were surveyed about their teamwork conditions and team process experiences. Overall, the tested model appears operational; however, the statistics suggest a marginal level of fit. These findings suggest that practical interventions focusing on specific team resilience dimensions—resourcefulness, shared goal alignment, proactive problem-solving robustness, capability to face difficulties—may support team effectiveness.

## 1. Introduction

Interdisciplinary teamwork is considered vital to ensure high-quality and safe care for people living with and beyond cancer [1,2]. Teamwork refers to the practice of organizing and integrating shifting multi-individual and multi-team sets and subsets with various backgrounds and expertise at different phases across the cancer care continuum [3,4]. Interdisciplinarity involves assembling insights from different providers and the patient to coordinate care along the cancer trajectory towards shared goals [5,6,7,8]. Interdisciplinary teamwork takes form adaptively; when providers manage to work toward shared goals despite difficulties, team cohesion emerges and benefits team functioning [9].

A team is deemed to be in a state of cohesion when its members develop bonds, share skills, and support each other in managing emotionally challenging situations. Evidence consistently identifies cohesion as key to high-functioning teams [10,11]. Team functioning, in turn, reflects affective, behavioral, and cognitive responses to face and manage difficult events and sustain team member well-being and high-quality care [12].

Multiple challenging acute or continued difficulties, also called adversity [13], are experienced in oncology care and can jeopardize team effectiveness [14,15,16]. Team resilience at work may influence the relationships between the teamwork practice environment and team functioning and cohesion, both of which are markers of team effectiveness [17]. Understanding this relationship is particularly relevant in oncology, where persistent clinical, emotional, and organizational demands challenge teams’ capacity to deliver high-quality care.

## 2. Background

Effective teamwork benefits patients by reducing hospitalizations, readmissions, morbidity, mortality and out-of-pocket costs, while improving health-related quality of life, person-centered care, and communication [2]. Higher-intensity interdisciplinary teamwork also contributes to prompt access to care, better patient-professional communication, person-centeredness and continuity of care [18]. From a workforce perspective, robust team functioning reduces burnout, enhances productivity and work satisfaction, promotes mental health and fosters a sense of coherence at work [2]. Conversely, compromised teamwork contributes to medical errors, lower provider and patient satisfaction, lower workforce retention, system inefficiencies and increased system costs [1,19].

Oncology teams in many jurisdictions confront challenges related to clinical care delivery, administrative burdens and a shrinking healthcare workforce [20,21,22,23] alongside pressures created by rapid treatment development, unique and evolving patient needs and a preoccupation with cost control. This atmosphere of perpetual crisis in healthcare systems [6,15] contributes to professional exhaustion, compassion fatigue, and loss of a sense of coherence [14,21,22,24,25,26,27,28]. Additional sources of adversity include the growing prevalence of specialty-based practice [29], along with tensions around professional role differentiation, both of which are associated with burnout in oncology team members [14,21]. These adversities underscore the need to better understand how team resilience influences team functioning and cohesion [30,31,32,33,34,35].

Recent attention to teamwork science, including two special issues of the Journal of Oncology Practice [36,37,38], highlights evolving evidence on team structures and processes that support high-quality multidisciplinary cancer care and emphasize the need for evidence-based interventions to strengthen teamwork and care coordination. However, resilience, as a teamwork process, remains under-addressed. The resources and mechanisms that enable resilient teams to maintain functioning and cohesion in especially challenging times (e.g., the COVID-19 pandemic and its aftermath) have not been studied extensively. This represents an opportunity to pay more attention to the role of team resilience at work in oncology to cope with multi-faceted sources of adversity.

Team resilience has caught the interest of researchers across disciplines over the past two decades [34]. Literature reviews and selected bibliographies provide a general understanding of this multidimensional concept and its potential impact [30,31,33,35,39]. In the present study, team resilience is conceptualized as the oncology team’s ability to cope with daily work pressures while preserving its health and well-being, adapting to change, and proactively preparing for upcoming crises, shocks, or stressors [40]. Resilient teams manage to function well despite difficult situations, enhance their capacity to minimize the negative effects of adversity and prepare for the future [41,42]. Empirical studies that have examined team resilience in oncology [24,43,44,45,46] employ heterogeneous research designs and data collection tools, limiting the ability to draw conclusions and hindering the translation of research into practice or decision-making.

This study explores the mediating role of team resilience at work on the relationships between the teamwork practice environment, team functioning and cohesion in oncology.

## 3. Materials and Methods

### 3.1. Theoretical Framework

**Figure 1:**
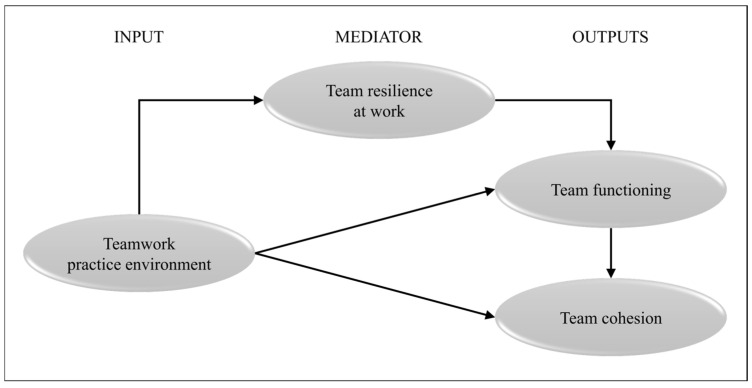
Input–mediator–output theoretical framework. Source: developed by the research team for this study.

The present study is guided by an input-mediator-output theoretical framework developed by the research team to probe deeper into relationships drawn from the literature [47]. The work environment constitutes the contextual antecedent shaping oncology team conditions. Team resilience at work functions as a mediating adaptive capacity describing how teams monitor, manage, and recover from adversity. Team functioning and team cohesion are distinct outcomes: functioning reflects day-to-day operational effectiveness, whereas cohesion captures the relative stability of psychological and social bonds within the team. Together, these concepts clarify how oncology teams sustain effectiveness under routine and adverse conditions. Figure 1 illustrates the relationships between the input (teamwork practice environment as independent variable), the mediator variable (team resilience at work), and outputs (team functioning, team cohesion as dependent variables). This framework postulates that the perceived teamwork practice environment influences team resilience at work, which in turn mediates team functioning and cohesion. It informs the structure of the statistical analysis, allowing for the empirical assessment of indirect effects and overall model fit within real-world oncology settings.

### 3.2. Study Design

This study is conducted within the context of a larger research project using a multi-component intervention aimed at better understanding the factors and mechanisms at play in team resilience at work in oncology [43]. A cross-sectional design is used to examine the relationships proposed in our theoretical framework by surveying oncology team members at a single time point [48].

### 3.3. Participants, Eligibility Criteria and Sample Size

Participants were members of oncology teams nested within the oncology departments of four hospitals in the national cancer network. Teams included direct-care professionals [49], namely clinicians (e.g., oncologists, nurses, pharmacists, social workers, psychologists, physical therapists, and nutritionists), managers and clerical staff. Team composition and size were comparable across hospitals, consistent with Québec Cancer Program guidelines [50]. The network-based configuration provides structural conditions that facilitate relational and cognitive proximity, both of which are central to interdisciplinary team-based care [51].

Convenience sampling was used to identify potential participants according to the following eligibility criteria: being a regular member of an oncology team; working at least 20 h per week to have sufficient exposure to teaming processes; and having worked in the oncology team without long-term leave during the 12 months before data collection. A total of 189 participants returned fully completed questionnaires. Sample size adequacy was confirmed using structural equation modeling (SEM) power calculation [52,53]. With a Root Mean Square Error of Approximation (RMSEA) of 0.09 as the alternative hypothesis vs. 0.05 as the null hypothesis and degrees of freedom of 537, a risk α of 0.05, we obtain a power of 1 − β = 0.99, supporting sufficient statistical power for the tested model.

### 3.4. Data Collection Measures

Self-administered e-questionnaires included a set of French-language validated instruments adapted to the oncology teamwork environment. Instruments were selected for their psychometric properties and low respondent burden. The e-questionnaire included a section for sociodemographic information. Table 1 presents the variables and subscales, along with descriptors relevant to oncology settings.

Teamwork practice environment is operationalized using Lurie and colleagues’ checklist (Mini-PEC), including items on environment suitable to accomplishment, availability of information needed to perform tasks and the possibility of sharing ideas, efforts to understand problems, and ability to act on the team vision [56]. The Mini-PEC informs on how team members interact with each other. It has 5 items with Cronbach’s alpha of 0.82 on a 4-point scale ranging from 1 = strongly disagree to 4 = strongly agree. This checklist could be completed in a very short time, increasing its feasibility for professionals with heavy workloads [57].

Team resilience at work is operationalized with the French-language version of McEwen and Boyd’s R@W Team Scale (TR@W Scale) [40]. The scale includes 42 items rated on a 7-point scale (1 = strongly disagree, 7 = strongly agree). Items are grouped into seven subscales: Resourcefulness, Robustness, Self-care, Alignment, Capability, Connectedness, Perseverance. Higher scores correspond to greater team resilience at work. This scale was selected as it conceptualizes team resilience as a collective capacity rather than the sum of individual traits [40,58] and recognizes that resources emerge and are mobilized differently in resilient teams than in resilient individuals [35]. Previous use in Québec showed acceptable to excellent internal consistency, with Cronbach’s alpha ranging from 0.61 to 0.93 for subscales and at 0.96 for the full scale, and means similar to the original scale [59].

Team functioning is operationalized using a subscale developed by Rousseau and colleagues [54]. The instrument assesses observable teamwork behaviors that facilitate collective task accomplishment from an operating perspective [60]. The items focus on back-up behaviors, information exchanges, psychological support and collaborative problem-solving, emphasizing team member participation in a collaborative practice. The subscale includes 12 items rated on a 5-point scale (1 = not true; 5 = totally true) and shows a high internal consistency with Cronbach’s alpha of 0.84.

The Team Cohesion scale is derived from the Interdisciplinary Team Performance instrument developed by Temkin-Greener [55]. The 6-item subscale measures shared goals and team identity, rated on a 5-point scale (1 = strongly disagree, 5 = strongly agree). Strong team identity enables sense-making in times of adversity [58].

Sociodemographic variables align with Cloconi et al. [61] and include age, gender, education level, type of profession, professional experience, professional experience in the oncology team, workweek hours and professional role.

### 3.5. Procedures

Following approval from the research ethics board, the principal investigator presented the study to potential participants during regular oncology team meetings. This pre-notification with operational information aimed to prepare data collection, answer questions related to expected time investment and encourage participation [62]. A designated local representative was involved in participant recruitment, collaborating with a research professional who verified participant eligibility. The local representative, an employee familiar with the oncology team members, collaborated with the research professional to validate inclusion and exclusion criteria. The research professional had over five years of experience in applied research in primary care and oncology settings, as well as prior experience in integrated knowledge mobilization. Eligible participants were then sent a personalized invitation with a unique login link to access the e-questionnaire, followed by two reminders 7 and 14 days later. Participation was voluntary. After reading the e-consent form presented on the first page, participants indicated their agreement electronically before proceeding. Responses were anonymous and collected via an individual SurveyMonkey account, ensuring confidentiality. Data collection began on 21 February 2022, and was completed on 19 June 2023.

### 3.6. Statistical Analysis

Data collected through the SurveyMonkey platform were exported to an Excel table using the survey dataset. Statistical analyses were performed using SAS software (version 9.4) [63]. Missing data were handled using listwise deletion without compromising statistical power given the adequate sample size. Statistical significance was set at *p* < 0.05. Descriptive statistics (mean and standard deviation (SD)) were used to summarize the variables and examine distribution. Standardized Cronbach’s alpha was used to determine internal consistency for the variables. Bivariate Pearson correlational analyses were examined to verify associations between input, mediator and output variables.

We performed SEM to assess the alignment between our input-mediator-output framework (Figure 1) and empirical data [64]. All constructs were modeled as latent variables, with sociodemographic data as covariates. Structural equation modeling (SEM) was performed to assess the fit between the proposed input–mediator–output conceptual framework (Figure 1) and the empirical data [64]. All constructs were specified as latent variables, with sociodemographic characteristics included as covariates. Associations among variables were estimated using standardized β coefficients. Model fit was evaluated using the Root Mean Square Error of Approximation (RMSEA < 0.08), Standardized Root Mean Square Residual (SRMR < 0.08), Comparative Fit Index (CFI > 0.90), Goodness-of-Fit Index (GFI > 0.90), and Tucker–Lewis Index (TLI > 0.90), in accordance with conventional cut-off criteria [52,65,66].

## 4. Results

### 4.1. Response Rate and Participant Characteristics

Among eligible participants, 26% returned the questionnaire. After excluding partially completed surveys (>20% missing), 189 questionnaires were retained for analysis. Table 2 summarizes participant characteristics. Most respondents were female (80.42%), and nurses represented the largest professional group (39.26%). Nearly 40% of participants reported ≥10 years of oncology experience. Roles varied: 43.4% provided direct clinical care, 34.9% held dual clinical and managerial responsibilities, and 7.2% were managers. Administrative and clerical personnel accounted for 14.5%. The mean age was 42.6 years (SD = 10.3), and the average workweek was 38.5 h.

### 4.2. Descriptive Statistics and Correlational Analysis

Table 3 presents descriptive statistics and bivariate correlations. Teamwork practice environment presented an acceptable level (mean = 3.03; SD = 0.56) and team resilience at work was high (mean = 4.88; SD = 1.09). Both team functioning (mean = 3.63; SD = 0.75) and team cohesion (mean = 3.95; SD = 0.82) were also high. Correlation coefficient results show a positive and significant correlation between teamwork practice environment and team functioning (r = 0.78; *p* < 0.0001) and team cohesion (r = 0.66; *p* < 0.0001). These two variables also have a significant and positive direct relationship with each other (r = 0.65; *p* < 0.0001). Correlation coefficients r > 0.50 suggest moderate to strong associations with high statistical significance, consistent with guidelines [67].

### 4.3. Model Fit

**Figure 2:**
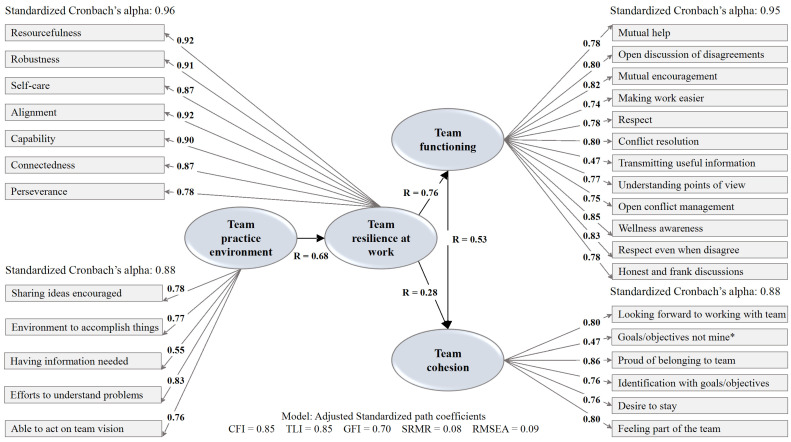
Team resilience at work mediating role model. * Reverse scoring for negatively phrased item.

The model presented in Figure 2 shows that all variables have had an impact with standardized Cronbach’s alpha ranging from good to excellent, ranging from 0.88 to 0.96 [68]. The highest impact was for team resilience at work (standardized Cronbach’s alpha = 0.96), especially resourcefulness and alignment variables. The item best at explaining the teamwork practice environment was effort to understand problems (R = 0.83) while the item that best explains team functioning was wellness awareness (R = 0.85); pride of belonging was best at explaining team cohesion (R = 0.86). Results suggest a significant positive direct effect of teamwork practice environment on team resilience at work (R = 0.68) which in turn had the strongest positive relationship with team functioning (R = 0.76); its relationship with team cohesion was weaker (R = 0.28). Structural equation modeling reflects the following model fit indices: CFI = 0.85, TLI = 0.85, GFI = 0.70, SRMR = 0.08, RMSEA = 0.09. The CFI and TLI values are slightly below commonly used thresholds, while the RMSEA is within a range indicative of marginal fit.

## 5. Discussion

The study examined a model in which team resilience at work is positioned as a potential mediator in the relationship between the team practice environment and team functioning and cohesion in oncology settings. Overall, the tested model appears operational; however, the fit indices suggest a marginal level of fit. While some indices approach commonly accepted thresholds, others are below recommended standards, indicating that the specified model does not fully capture the underlying covariance structure. Taken together, these results support the model as a preliminary framework, while also indicating that further refinements such as structural adjustments, the addition or removal of variables, or revisions to covariances could improve its adequacy and theoretical alignment.

The observed convergence between research evidence and real-world data underscores the relevance of keeping interdisciplinary teamwork on the agenda of organizational stakeholders [4,5,69,70]. Team resilience at work may contribute to a better understanding of the intrinsic functioning of oncology teams and appears as a general resistance resource in difficult times. Although previous research has documented a direct association between the teamwork practice environment and team functioning [16,22], the present findings suggest that incorporating team resilience at work as a mediator may offer additional insight into potential targets for improvement around dimensions especially relevant in oncology settings.

There is also evidence outlining actionable steps that could support team effectiveness. For example, core components of teamwork include mutual performance monitoring, back-up behaviors, adaptability and team orientation [71]. In oncology, these demanding mechanisms rest on the recognition of interdependencies in fragmented health systems. Efforts to reduce siloed functioning may therefore benefit from deliberately creating cognitive and relational proximity [51], mutual trust, closed-loop communication, shared mental models and coordination [72]. These processes are thought to activate within-team mechanisms to maintain cohesion. In turn, the resulting co-constructed knowledge may contribute to team resilience capacity which could help sustain functioning and cohesion in the delivery of high-quality care and a whole-person centered response [23,73,74].

A noteworthy finding of this study relates to the latent dimensions identified through the modeling process. Resourcefulness, alignment, efforts to understand problems, wellness awareness and being proud to work in the team emerged as salient team-level elements. These items may inform management practices and point to targeted interventions aligned with resilience-building efforts to bounce beyond difficult situations. From this perspective, developing team agility to juggle the volatility, uncertainty, complexity, and ambiguity (VUCA) [75] characterizes the challenges of the teamwork practice environment in oncology. While strategies at the organizational and system levels to improve staff supply in specialized care, interdisciplinary training and patient partnership, and virtual technologies are expected to yield positive effects over time [20,76], team-level interventions may offer immediate support. For example, initiatives introduced during the COVID-19 pandemic, including regular educational communications and access to mindfulness and resilience-focused resources, have been associated with improved team cohesion [77].

Limitations should be kept in mind when interpreting the results of the present study. First, the response rate of 26% is consistent with rates commonly reported in electronic surveys of healthcare professionals. A meta-analysis by Cho et al. (2013) estimated that an acceptable overall response rate among clinicians (physicians, nurses, other health professionals) was 53% but may range from 9.1% to 72.1% depending on data collection mode, type of participant or number of reminders [78]. Although the participation rate was modest, the sample includes diverse team members from multiple institutions, supporting the relevance of the findings. Nevertheless, potential convenience sampling bias is acknowledged. The possibility that survey results are influenced by nonresponse invites us to be cautious about potential bias [79]. Second, selecting teamwork practice environment as the primary antecedent of team functioning and cohesion (effectiveness) may overlook other contextual or organizational determinants that could influence these relationships. Although data collection occurred over a one-year period, the cross-sectional design does not consider temporal variability. Changes that may have occurred within the data collection period were not controlled, which may present a limitation. Had we considered other determinant factors in the larger environment or team member turnover, results may have been different. Third, reliance on self-reported measures is prone to bias, as participants tend to provide socially desirable responses. Bias may have been introduced if respondents’ moods or their perceptions of team or organizational factors influenced the way they answered the questionnaire items [48]. Fourth, while SEM reduces the risk of measurement error [80], the marginal model fit indicates that observed associations are compatible with mediation but do not prove causal pathways [64] particularly given the cross-sectional design [80]. Finally, the generalizability of findings should consider that the teams participating in this study were embedded in a publicly funded system.

Despite these limitations, this study provides novel evidence suggesting that team resilience at work may be meaningfully associated with both operational and relational aspects in oncology settings. By identifying resilience-related dimensions that are potentially amenable to intervention, the findings complement existing work on teamwork effectiveness [1,3,37,69,74] and highlight the value of examining broader team processes beyond tumor-specific teams [69] or multidisciplinary treatment meetings [5].

Future research using longitudinal and experimental designs could provide additional insights into the temporal and directional relationship between team resilience and teamwork effectiveness, and its mediating role with factors other than the practice environment. Future studies could use more detailed instruments that look at additional features of the practice environment that may enhance team resilience capacity. While the quantitative data analyzed in the study provides new insights on the mediating role of team resilience at work, qualitative data could further allow for a more comprehensive understanding of how to activate team resilience mechanisms in particular environments and plan tailored interventions that meet the needs of oncology teams. Given that mechanisms involved in team resilience at work may differ locally or regionally, additional research could explore the relationship between resilience at the organizational and cancer system levels [58,81].

## 6. Conclusions

This study adds to the growing body of literature examining how team resilience may be associated with effective teamwork in oncology settings. By identifying an indirect association compatible with a mediating role between the practice environment and team functioning and cohesion, our findings suggest the relevance of supporting resilience at the team level as oncology services face persistent organizational and clinical pressures. The results point to the value of team-level resources—such as alignment, resourcefulness, and wellness awareness—that help professionals navigate adversity while maintaining high-quality care. In addition, the study provides a replicable theory-informed approach for examining resilience-related mechanisms in real-world cancer care contexts. Future longitudinal or mixed-methods research is warranted to clarify how team resilience develops over time and informs interventions aimed at strengthening oncology teams’ capacity to adapt and perform under challenging circumstances.

## Figures and Tables

**Table 1:** Description and internal consistency (Cronbach’s alpha) of variables evaluated.

Variables and Subscales	Descriptors		Cronbach’s α	
		Nb of Items	Theoretical	Study
Team Resilience at Work (TR@W Scale) [40]		42	0.95	0.96
Resourcefulness	Optimizing oncology team strengths and available resources. Concentrating on continuous improvement. Activating team mechanisms geared toward priorities.	10	0.93	0.92
Robustness	Upholding shared representation and objectives. Demonstrating adaptability and a proactive approach to problem-solving.	8	0.85	0.91
Self-care	Encouraging stress management techniques and recognizing signs of work overload. Fostering a balance between work and personal life.	7	0.87	0.87
Alignment	Advancing towards shared goals. Maintaining a positive mindset. Recognizing milestones and achievements.	5	0.88	0.92
Capability	Achieving results in a changing environment.	7	0.89	0.90
Connectedness	Collaborating, supporting one another, and nurturing a sense of community within and between teams.	2	0.81	0.87
Perseverance	Mobilizing energies where they can make a difference, with a focus on solutions rather than problems.	3	0.83	0.78
Team Functioning (Team Functioning Scale) [54]				
Team functioning	Providing interpersonal support: Respecting one another, exchanging work-related insights, managing conflicts, and providing back-up to colleagues (operational perspective).	12	0.84	0.95
Team Cohesion (Interdisciplinary Team Performance Scale) [55]				
Team cohesion	Having a sense of belonging to the oncology team and adhering to team vision and objectives (relational perspective).	6	0.86	0.88
Team Practice Environment (Mini-PEC Checklist) [56]				
Team practice environment	Exchanging ideas, creating an environment conducive to success, ensuring access to necessary information, striving to comprehend challenges, and acting in accordance with the team’s vision.	5	0.82	0.88

**Table 2:** Participant characteristics (n = 189).

Variables	Mean ± SD	n ^1^	%
Age (years)	42.63 ± 10.28		
Gender			
Female		152	80.42
Other		37	19.04
Education			
Secondary school ^2^		10	7.87
College		39	30.71
University		78	61.41
Profession			
Cancer specialist (physician)		21	12.88
Nurse		64	39.26
Other team members (professional) ^3^		56	34.36
Support staff		15	9.20
Other		7	4.29
Role in oncology			
Clinical role in direct care		66	43.42
Management at front line and direction levels		11	7.24
Clinical + management combined		53	34.87
Administrative or other		22	14.48
Experience in oncology (years)			
<10		75	59.52
≥10		51	40.48
Average workweek hours	38.53 ± 8.60		

SD, standard deviation. ^1^ Valid responses only. ^2^ Support staff only. ^3^ Other team members (professional): professionals specialized in nutrition, pharmacy, physical therapy, psychology, social work.

**Table 3:** Descriptive statistics and bivariate Pearson correlations between team practice environment, team resilience at work, team functioning, team cohesion.

Variables (Min-Max Range)	Mean	SD	1	2	3	4
1. Team practice environment (1–4)	3.03	0.56	1.00			
2. Team resilience at work (1–7)	4.88	1.09	0.72 ***	1.00		
3. Team functioning (1–5)	3.63	0.75	0.78 ***	0.76 ***	1.00	
4. Team cohesion (1–5)	3.95	0.82	0.66 ***	0.65 ***	0.66 ***	1.00

SD, standard deviation. *** *p* < 0.0001. Note: Reverse scoring for negatively phrased item.

## Data Availability

The data presented in this study are available on request from the corresponding author due to ethical restrictions.

## Abbreviations

The following abbreviations are used in this manuscript:

CFI	Comparative Fit Index
GFI	Goodness of Fit Index
RMSEA	Root Mean Square Error of Approximation
SD	Standard deviation
SEM	Structural Equation Modeling
SRMR	Standardized Root Mean Squared Residual
TLI	Tucker–Lewis Index
TR@W	Team resilience at work
